# A Model for Cell Proliferation in a Developing Organism

**DOI:** 10.1007/s00285-022-01769-5

**Published:** 2022-06-25

**Authors:** Philip K. Pollett, Laleh Tafakori, Peter G. Taylor

**Affiliations:** 1 School of Mathematics and Physics, University of Queensland, Brisbane, Australia; 2 Department of Mathematical Sciences, RMIT University, Melbourne, Australia; 3 School of Mathematics and Statistics, University of Melbourne, Melbourne, Australia

**Keywords:** Continuous-time Markov chains, Proliferation processes, Yule-Furry processes, Stochastic models, 92B05 General biology and biomathematics, 60F05 Central limit and other weak theorems

## Abstract

In mathematical biology, there is a great deal of interest in producing continuum models by scaling discrete agent-based models governed by local stochastic rules. We discuss a particular example of this approach: a model for the proliferation of neural crest cells that can help us understand the development of Hirschprung’s disease, a potentially-fatal condition in which the enteric nervous system of a new-born child does not extend all the way through the intestine and colon. Our starting point is a discrete-state, continuous-time Markov chain model proposed by Hywood et al. (2013a) for the location of the neural crest cells that make up the enteric nervous system. Hywood et al. (2013a) scaled their model to derive an approximate second order partial differential equation describing how the limiting expected number of neural crest cells evolve in space and time. In contrast, we exploit the relationship between the above-mentioned Markov chain model and the well-known Yule-Furry process to derive the exact form of the scaled version of the process. Furthermore, we provide expressions for other features of the domain agent occupancy process, such as the variance of the marginal occupancy at a particular site, the distribution of the number of agents that are yet to reach a given site and a stochastic description of the process itself.

## Introduction

Cell motility and cell proliferation play an indispensable role in collective cell spreading, which is essential to many key biological processes, medicine and pathological mechanisms including foetal tissue growth, tumour growth and wound healing (see e.g., Maini et al. 2004a; Deng et al. 2006; Cai et al. 2007; Friedl and Gilmour 2009), cell mobility in orchestrating morphogenesis during embryonic development (Gilbert 2003; Keller 2005; Binder et al. 2008; Vulin et al. 2014), immune responses Madri and Graesser (2000), tissue domain expansion (Crampin, Hackborn and Maini 2002), cancer Weinberg and Hanahan (2000) and vascular disease Raines (2000).

Cell proliferation and cell division process lead to domain expansion. This can change the cellular spatial distribution and consequently lead to mechanisms of transport for proliferating cells. Understanding this mechanism in controlling collective cell migration can, potentially, enable us to prevent any abnormalities such as developmental anomalies or cancer metastasis. Agent-based cellular models have widely been used to model proliferative tissue growth (Binder and Landman 2009; Hywood et al. 2013b; Ross et al. 2016). The methods that are typically used to develop these models are based on using a master equation or by using the Fokker-Planck partial differential equation approach, Pillay et al. (2017). Applications of using these developed mathematical models have extensively been used for bone and tumour growth (Czarnecki et al. 2014; Monteagudo and Santos 2015), embryonic tissue growth by assuming time evolution of the length of the tissue domain was known (Binder et al. 2008; De Oliveira and Binder 2019) and fungal and yeast colonies (Matsuura 2000; Tronnolone et al. 2017). Therefore, to improve our understanding of proliferation cell processes, mathematical models have been formulated for the behaviour of the cell population as a whole, based on the behaviour of the individual cells within the population. For example, in the model for the growth of the foetal gut proposed by Hywood et al. (2013a), neural crest cells populate the gut during its growth and remain in the gut tissue to form the neurons that construct the enteric nervous system (ENS). Failure of the neural crest cells to invade the gut tissue completely can cause imperfect formation of the ENS, and lead to the existence of Hirschsprung’s disease Newgreen and Young (2002).

According to Grosfeld (2008), the first report of this disease may have occurred back in 1691. However, the disease is named after the Danish pediatrician Hirschsprung (1981), who described it. This disease can result in serious colon complications, such as enterocolitis and toxic megacolon, which can be life threatening, and it is vital that it be diagnosed early so that medical care can be commenced in a timely manner.

In the literature, there are two main approaches to explaining collective cell spreading: (i) discrete models, and (ii) continuum models Murray (2012). The first class, discrete, individual-based models, usually involve discretizing time and/or space. These models capture the stochasticity and nonuniformity observed in experiments Simpson et al. (2007). The benefit of these models is that they can predict features based on information Murray et al. (2011) and fluctuation Simpson et al. (2014) that can be measured in experimental data Simpson et al. (2010).

In parallel to discrete models, much attention has also been given to developing continuum models, illustrating the behaviour of populations of cells using reaction-diffusion equations. Continuum models can be used to clarify the relationships among the model parameters so that they can be investigated numerically Maini et al. (2004b). The interplay between pattern formation and domain growth, studied in Crampin, Gaffney and Maini (2002), and Woolley et al. (2011), makes use of both deterministic and stochastic aspects.

A multiscale conception of the complex processes driving cell migration can be attained by linking these two modelling approaches together in an equivalent framework; this gives intuition into the interplay between the individual level and population level models that enable us to use either modelling scenario to study appropriate properties of the system. Hywood et al. (2013a) developed such a linkage by studying a discrete, stochastic, domain growth model where discrete ‘domain agents’ representing the gut cells proliferate. This model is a one dimensional semi-infinite lattice model for tissue growth with lattice spacing $\Delta$. Let *N*(*t*) be the number of domain agents at time *t* and $L(t) \equiv \Delta N(t)$ be the length of the gut at this time. It follows that $x=i\Delta$ is the position of the *i*th lattice site. In this model a proliferation event can occur, which results in the inclusion of an additional domain agent into the lattice. Consequently, the number of agents rises by one with each proliferation. In this process, if the domain agent at site *i* is selected to proliferate, it moves to site $i+1$ and pushes all domain agents in sites $i+1,i+2,\ldots$ one place higher.

**Fig. 1:**
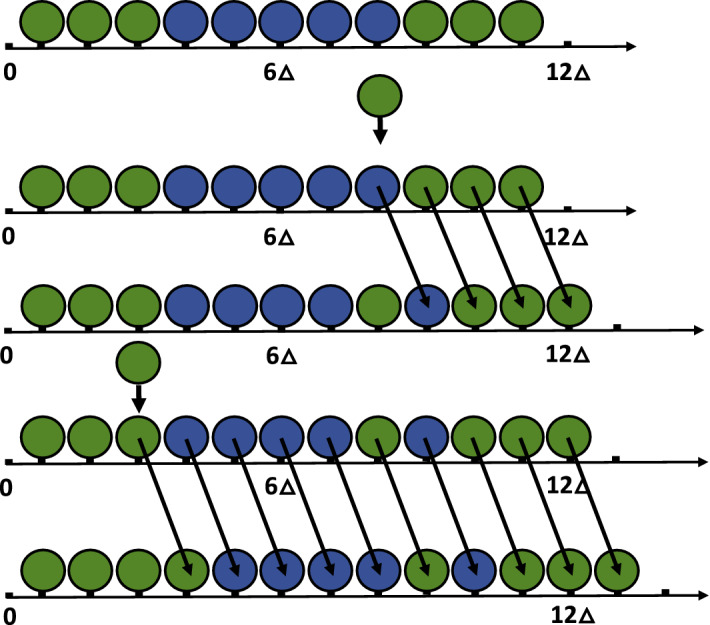
(Color online) A diagrammatic representation of a growing lattice made up of domain agents, some of which are marked (blue [dark gray]). Initially there are eleven domain agents in total. The domain agents occupying sites 4-8 are marked. The remaining are unmarked (colored green). The new unmarked domain agent is added to the lattice, occupying site 8. The arrows show the movements of respective domain agents. In the next step, the unmarked domain agent at site 3 is selected to proliferate. The new unmarked domain agent is added to the lattice, occupying site 3

This process is depicted in Fig. 1 which demonstrates a realization of two proliferation events with a subset of blue marked and green unmarked agents. Whenever a marked or an unmarked agent is selected to proliferate, it moves one site to the right and an unmarked domain agent is inserted into the lattice. If a marked domain agent is selected to proliferate, then the addition of the unmarked domain agent will not only extend the lattice but possibly also separate marked domain agents.

By letting the spacing $\Delta$ shrink to zero, Hywood et al. (2013a) derived a PDE (their Eq. (14)) to describe how the continuous expected occupancy evolves in time. The coefficient of the diffusion term in this PDE is a linear function of $\Delta$, and hence it should be taken to be zero as the lattice spacing shrinks. However, Hywood et al. (2013a) observed a very good fit between simulations and the trajectories of this PDE, when $\Delta$ is taken to be small and positive.

Our aim in this work is to analyse the model of Hywood et al. (2013a) in a different way, by observing that the proliferation process of Hywood et al. (2013a) can be interpreted as a superposition of Yule-Furry processes. We are able to derive expressions for the expectation and variance of the occupancy at a given point, the distribution of the number of agents yet to reach a given point and a stochastic description of the process itself.

## The Proliferation Process

In this paper we work on the proliferation process introduced by Hywood et al. (2013a). Let $\{X(t)\}_{t\ge 0}=\{(X_1(t), X_2(t),...,X_{N(t)}(t))\}_{t\ge 0}$ be a random vector that models the state of a proliferation process at time *t*, with *N*(*t*) the number of sites at time *t* and $X_i(t)=1$ if site *i* is occupied by a marked domain agent at time *t* and equal to zero otherwise. Let $T_k$ denote the time at which the *k*-th proliferation event occurs with $T_0=0$ and $\tau _{k+1}=T_{k+1}-T_k$ the time between the *k*th and $k+1$st events. We assume that the random variable $\tau _{k+1}$ is exponentially distributed with rate $\lambda N(T_{k}) = \lambda (N(0) + k)$, so that the expected value of $\tau _{k+1}$ is $1/(\lambda (N(0) + k))$, and that the location of the $k+1$st proliferation event has a discrete uniform distribution on $\{1,\ldots ,N(0) + k\}$. If the proliferation event occurs at site *j* then $X(T_{k+1})=(X_1(T_k),\ldots ,X_{j-1}(T_k),0,X_j(T_k),\ldots ,X_{N(T_k)}(T_k))$.

The above assumptions imply that the probability that a proliferation event occurs in $(t,t+\tau ]$, when the state is *X*(*t*), is $\lambda N(t) \tau + o(\tau )$ and the location of such a proliferation is to the left of site *i* with probability $(i-1)/N(T)$. Noting that $X_i(t+\tau ) = 0$ if a proliferation event occurs exactly at site *i*, it follows that the expected occupancy ${{\mathbb {E}}}(X_{i}(t+\tau )|X(t))$ of site *i* at time $t+\tau$ satisfies the equation

1 $$\begin{aligned} {{\mathbb {E}}}(X_{i}(t+\tau )|X(t))= & {} (1-\lambda N(t)\tau )X_{i}(t) \nonumber \\&+ \lambda N(t)\tau \left[ \left( \frac{i-1}{N(t)}\right) X_{i-1}(t)+ \left( 1-\frac{i}{N(t)}\right) X_{i}(t)\right] + o(\tau ).\nonumber \\ \end{aligned}$$

Taking expectations of both sides, we can derive an equation for $C_i(t) \equiv {{\mathbb {E}}}(X_{i}(t)|X(0))$,

2 $$\begin{aligned} C_i(t+\tau ) = C_{i}(t) + \lambda \tau \left( (i-1) C_{i-1}(t) - i C_{i}(t)\right) + o(\tau ). \end{aligned}$$

We can model an initial condition where there is a contiguous segment of lattice sites $r+1,\ldots ,s$ that are occupied by marked agents by taking $X_i(0) = C_i(0) = I(r < i \le s)$.

To derive a PDE, we make the transformation of variables $i\rightarrow \frac{x}{\Delta }$ and $C_{i}(t)$ to *C*(*x*, *t*), divide both sides of Eq. (2) by $\tau$, and take limits $\Delta \rightarrow 0$ and $\tau \rightarrow 0$ so that

3 $$\begin{aligned} \frac{\partial }{\partial t}C(x,t)=-\lambda \frac{\partial }{\partial x}\left( xC(x,t)\right) . \end{aligned}$$

If we want to take as an initial condition that $C(x,0) = 1$ on some interval (*a*, *b*], and zero otherwise, we need to let both *r* and *s* in the above model approach infinity in such a way that $\lim _{\Delta \rightarrow 0} r \Delta = a$ and $\lim _{\Delta \rightarrow 0} s \Delta = b$.

Equation (3) is valid for $0\le x < \widetilde{L}(t)$, with ${{\widetilde{L}}}(t)$ the length of the domain at time *t* in the continuous model. With $L(t)\equiv \Delta N(t)$, observing that, for any *t* and $\tau$ in the discrete model,

$$\begin{aligned} {{\mathbb {E}}}[L(t+\tau )|L(t)] = \left( 1-\lambda N(t)\tau \right) L(t)+ \lambda N(t)\tau \left( L(t)+ \Delta \right) + o(\tau ), \end{aligned}$$

it follows that ${{\mathbb {E}}}[L(t)|L(0)]$ satisfies the differential equation

4 $$\begin{aligned} \frac{d{{\mathbb {E}}}[L(t)|L(0)]}{dt} = \lambda {{\mathbb {E}}}[L(t)|L(0)], \end{aligned}$$

and so

5 $$\begin{aligned} {{\mathbb {E}}}[L(t)|L(0)] = L(0) e^{\lambda t}. \end{aligned}$$

We see that the expected length of the domain in the discrete model grows exponentially. Via the limiting procedure used above, this is also true of the length ${{\widetilde{L}}}(t)$ of the deterministic domain of the PDE (3).

Hywood et al. (2013a) proposed that the expected occupancy *C*(*x*, *t*) is well-modelled by the second-order partial differential equation.

6 $$\begin{aligned} \frac{\partial }{\partial t}C(x,t)=\frac{-L'(t)}{L(t)}\frac{\partial }{\partial x}\left( xC(x,t)\right) +\frac{L'(t)\Delta }{2L(t)}\frac{\partial ^2 }{\partial x^2}\left( xC(x,t)\right) . \end{aligned}$$

**Fig. 2:**
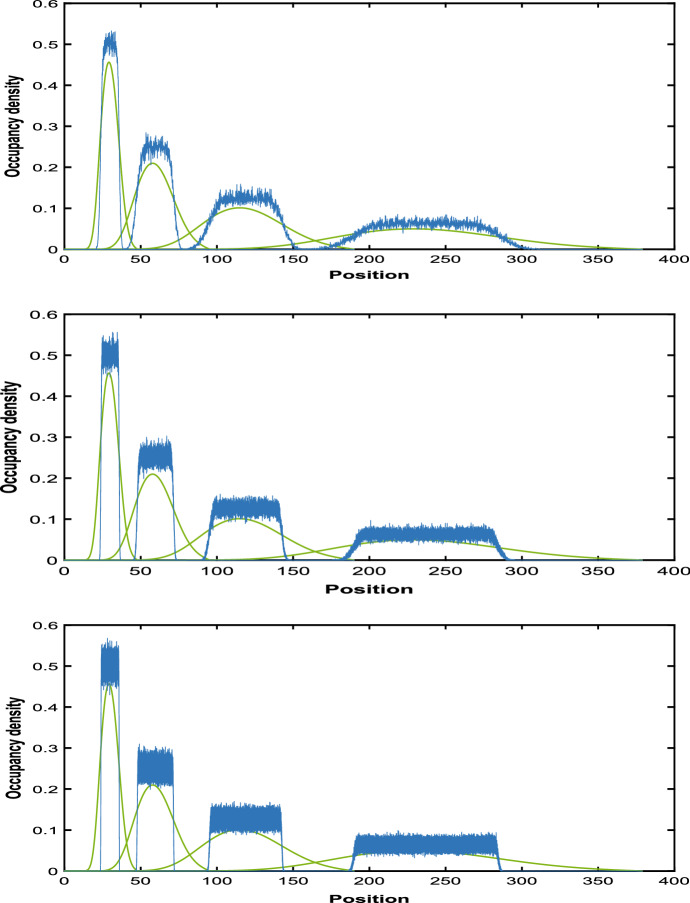
Expected occupancy averaged over 1000 realizations (blue) and Solutions to Eq. (6) (green curve) with $\Delta = 0.1$, $\Delta =0.01$ and $\Delta =0.001$ at times $t = 1.0, 2.0, 3.0, 4.0,$ with initial particle mass on the interval [12, 18] and $\lambda = 0.69$

Because of the form (5), the first order term on the right hand side of (6) is the same as that in (3). Hywood et al. (2013a) noted that the trajectories of Eq. (6) fitted simulated results well. However it is clear that the second order term on the right hand side should be zero, as $\Delta \rightarrow 0$. Indeed, the two limits $$\lim _{\begin{array}{c} \Delta \rightarrow 0 \\ \tau \rightarrow 0 \end{array}}\Delta ^2/\tau $$ and $$\lim _{\begin{array}{c} \Delta \rightarrow 0 \\ \tau \rightarrow 0 \end{array}}\Delta /\tau $$ required to derive (6) cannot both be finite and non-zero. Thus, this equation has to be regarded as an approximation. In Fig. 2, we compare Eq. (6) derived by Hywood et al. (2013a) with simulation results averaged over 1000 of realizations for small values of $\Delta$. We can see that it does not fit well.

In Sect. 3, we shall present an alternative view of the proliferation process that exploits the fact that individual particles move according to a Yule-Furry process. In the following Sect. 4, we shall scale this representation to derive the exact form of the continuum limit of the model. We provide the proofs of the main results in Sect. 5.

## Yule-Furry Processes and Proliferation Processes

The pure birth process with a Markovian structure introduced by McKendrick (1914), is considered as one of the simplest branching processes. In the case that the birth rate is linear, it is called the pure linear birth, classical Yule or Yule-Furry process see, for example, Yule (1925), Bharucha-Reid (1997), De La Fortelle (2006) and references therein. The Yule-Furry process has been used to model stochastic population growth with a preferential attachment process as a model of speciation in biology. It was developed with the aim to model various stochastic dynamical systems processes in different fields ranging from epidemics, demography and population biology to server queuing modelling Elia and Taricco (1992), mathematical biology Baake and Wakolbinger (2015) and branching processes Kendall (1966). For instance, Romero-Arias et al. (2018) analysed a growth model of an avascular tumour that considers the basic biological principles of proliferation and genetic mutations of the cell. They modelled the cell mutations dynamics by using a Yule-Furry process and by assuming that cell mutation depends only on the previous genetic state.

Mathematically, the *Yule* or *Yule-Furry* process is a pure-birth continuous-time Markov chain on the state space $\{1,2,\ldots \}$ with transition rates $q(i,i+1)=\lambda i$ for some $\lambda > 0$, which is known as the *splitting rate*, see for example (Taylor 1975, Page 122). It is well-known that if a Yule-Furry process starts in state *j* at time zero then its position at time *t* has a negative binomial distribution with parameters *j* and $e^{-\lambda t}$, that is the probability that it is in state $k\ge j$ at time *t* is

7 $$\begin{aligned} p_{jk}(t) = {{k-1}\atopwithdelims (){k-j}} e^{-j\lambda t}\left( 1-e^{-\lambda t}\right) ^{k-j}, \end{aligned}$$

with $$p_{jk}(t) = 0$$ if $$k<j$$.

A domain agent in the proliferation process of Hywood et al. (2013a) moves according to a Yule-Furry process. Observing that the number of domain agents $U_k(t)$ at a given position $k\ge 1$ at time *t* is necessarily 0 or 1, it easily follows that

8 $$\begin{aligned} E_j[U_k(t)]= & {} \Pr (Y(t)=k|Y(0)=j)\nonumber \\= & {} {\left\{ \begin{array}{ll}{\displaystyle {{k-1\atopwithdelims ()k-j}e^{-j\lambda t} \left( 1-e^{-\lambda t}\right) ^{k-j} }}&{}\quad \text {if}\;\ k\ge j\\ { {0}}&{}\quad \text {if}\;\ k < j. \ \end{array}\right. } \end{aligned}$$

Now we are interested in the expected total number of particles at position *k* at time *t*, therefore, we consider $s-r$ of these processes, $Y^{(j)}(t)$, independently evolving on the positive integers, with common splitting rate $\lambda$ and with different starting points $j=r+1,...,s$. Let the number of particles at position *k* at time *t* in the *j*th process be denoted by $U^{(j)}_{k} (t)$. Then the expected total number of particles at position *k* at time *t* is given by

9 $$\begin{aligned} C_{k}(t) \equiv \sum ^{s}_{j=r+1}E[U^{(j)}_{k}(t)]= \sum _{j=r+1}^{\min (k,s)} {k-1\atopwithdelims ()k-j}e^{-j\lambda t }(1-e^{-\lambda t})^{k-j}. \end{aligned}$$

An important observation, made by Hywood et al. (2013a), is that this expected total number of particles is given by (9) even if the processes $Y^{(j)}(t)$ are dependent, and that imposing a particular type of dependence results in the discrete space proliferation model described in Sect. 1.

To see this, observe that the position *Y*(*t*) at time *t* of a particle evolving according to a Yule-Furry Process with splitting rate $\lambda$ and with $Y (0) = j$ satisfies the stochastic equation

10 $$\begin{aligned} Y (t) = j + {{{\mathcal {N}}}}(\Lambda (t)), \end{aligned}$$

where ${{\mathcal {N}}}(\Lambda (t))$ is the number of points in an inhomogeneous Poisson process with an intensity measure depending on the evolution of *Y* on the interval [0, *t*) given by

11 $$\begin{aligned} \Lambda (t) \equiv \Lambda [(0,t)]=\int ^{t}_{0}\lambda Y(u)du. \end{aligned}$$

**Fig. 3:**
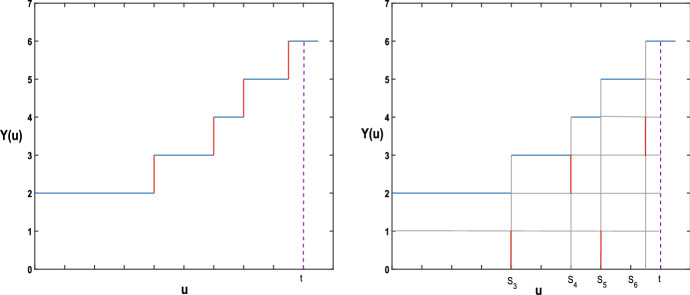
Left-hand panel: the integral (11) evaluated with with respect to *t*. The jumps occur at exponential intervals with increasing rate. Right-hand panel: the integral (11) evaluated with respect to *Y*. The jumps are triggered by events in the Poisson processes ${{\mathcal {N}}}_1$, ${{\mathcal {N}}}_3$, ${{\mathcal {N}}}_1$ and ${{\mathcal {N}}}_4$ respectively

To simulate a Yule-Furry process according to this viewpoint, we could use the fact that the integrand in (11) is piecewise constant and so, when $Y(t) = k$, we draw an exponential random variable with parameter $k \lambda$ and increase the state to $k+1$ when this time has elapsed.(Fig. 3)

Another way to simulate the process would be to generate independent homogeneous Poisson processes $\{{{\mathcal {N}}}_{i}\}$ with rate $\lambda$ at each integer point *i* and move the particle at *k* one place to the right at any instant when there is an event in one of the Poisson processes ${{\mathcal {N}}}_i$ with $i\le k$. This is equivalent to thinking of the random intensity measure in (11) as generated by the independent homogeneous Poisson processes $\{{{\mathcal {N}}}_{i}\}$. Specifically, by expressing the area represented by the integral in (11) as a sum of the areas of horizontal rectangles, we see that

12 $$\begin{aligned} \Lambda [(0,t)]=\sum ^{Y(t)}_{i=1}\lambda (t-S_i), \end{aligned}$$

with $S_{i}=0$ for $i \le j$ and $S_i=\inf \{u:Y(u)\ge i\}$ for $i >j$. These two viewpoints are illustrated in Fig. 3 for a case where $j=2$. In the left hand panel the jumps are events in a Poisson process of increasing intensity. In the right hand panel, they are events in the superposition of an increasing number of Poisson processes.

Now let’s return to the $s-r$ processes $Y^{(j)}(t)$ driving Eq. (9). In the spirit of (12), we can think of each of these as being generated by a sequence $\{\mathcal{N}^{(j)}_{i}\}$ of Poisson processes, the *i*th process corresponding to the integer point *i*. If these processes are independent for different values of *j*, then $C_{k}(t)$ is the *asymmetric random walk process* of Hywood et al. (2013a).

However, if the processes are identical so that $\{\mathcal{N}^{(j)}_{i}\}=\{{{\mathcal {N}}}_{i}\}$ for all *j*, then the model is the proliferation process of Hywood et al. (2013a): points in the *i*th Poisson process correspond to proliferation events at the *i*th site and move particles from all processes $Y^{(j)}(t)$ that are to the right of *i* simultaneously. In this way, the proliferation process can be regarded as a *coupled version* of the asymmetric random walk. This leads us to the following theorem.

### Theorem 3.1

Let $\{X(t)\}_{t\ge 0}=\{(X_1(t), X_2(t),...,X_{N(t)}(t))\}_{t\ge 0}$ be a random vector that models the state of a proliferation process as defined in Sect. 2 at time *t*, with *N*(*t*) the number of sites at time *t* and $X_i(0) = I(r < i \le s)$. Then

(i) The expected number of points at site *k* in the proliferation process is given by $C_k(t)$ in (9).
(ii) The variance in the number of points at site *k* in the proliferation process is given by $C_k(t)(1-C_k(t))$.
(iii) For $j=r,\ldots ,s-1$, let $Z^{(j)}(t)= Y^{(j+1)}(t) - Y^{(j)}(t)$, with $Y^{(0)}(t) = r$, so that $Z^{(j)}(t)$ measures the distance between the *j*th and $(j+1)$st marked domain agents at time *t*. Then the $s-r$ random variables $Z^{(j)}(t)$ are independent with common probability mass function given by
  13 $$\begin{aligned} f_Z(t) \equiv \Pr (Z^{(j)}(t)=k) = e^{-\lambda t}\left( 1-e^{-\lambda t}\right) ^{k-1}. \end{aligned}$$
(iv) For $k>r$, let $M_k(t) = \sum _{\ell =r+1}^k X_{\ell }(t)$ be the number of marked domain agents in positions $r+1,\ldots ,k$ at time *t*. Then
  $$\begin{aligned}&\Pr (M_k(t) \le j-r)\nonumber \\&\quad = {\left\{ \begin{array}{ll} \displaystyle 1 &{} \quad \text {if}\;\ k \le j\\ \displaystyle {k\atopwithdelims ()j} e^{-j\lambda t}\left( 1-e^{-\lambda t}\right) ^{k-j}\ _2F_1(k+1,1;k-j+1;1-e^{-\lambda t})&{}\quad \text {if}\;\ k > j. \ \end{array}\right. } \end{aligned}$$

where $_2F_1(a,b;c;z)$ is the hypergeometric function (Abramowitz and Stegun 1965, Definition 15.1.1).

### Proof

See the Appendix. $\square$

**Fig. 4:**
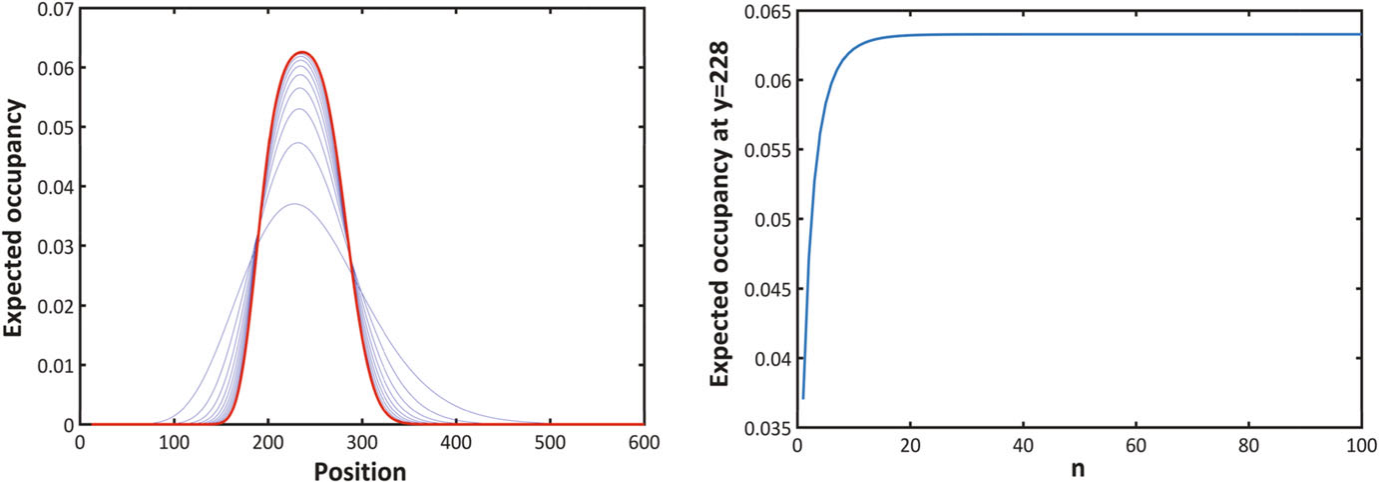
Left-hand panel: values of $C_{\Delta }(y, t)$ at $t = 4$ with initial particle mass on the interval [12, 18]. Right-hand panel: $C_{1/n}(228,4)$ plotted against *n*, $(\lambda = 0.69)$

## Asymptotic Properties

With a view to establishing a continuum limit of the model in Sect. 3, we suppose that there are $N(0) = {\left\lfloor {(b-a)/\Delta }\right\rfloor }$ particles initially situated at positions

$$\begin{aligned} \Delta ({\left\lfloor {a/\Delta }\right\rfloor }+1),\cdots ,\Delta {\left\lfloor {b/\Delta }\right\rfloor } \end{aligned}$$

within the interval (*a*, *b*] with equal spacing $\Delta$. The idea is that there is an initial ’particle mass’ $\Delta {\left\lfloor {(b-a)/\Delta }\right\rfloor }$ distributed evenly among these *N*(0) points. The point at position $\Delta ({\left\lfloor {a/\Delta }\right\rfloor }+j)$ carries a mass $\Delta$, which we can think of as a rectangle of unit height over the interval $[\Delta ({\left\lfloor {a/\Delta }\right\rfloor }+j),\Delta ({\left\lfloor {a/\Delta }\right\rfloor }+j+1)]$.

The expected height of the rectangle at position *y* at time *t*,

14 $$\begin{aligned} C_{\Delta }(y, t)= & {} \sum ^{{\left\lfloor {a/\Delta }\right\rfloor }+N(0)}_{j={\left\lfloor {a/\Delta }\right\rfloor }+1}E[U^{(j)}_{{\left\lfloor {y/\Delta }\right\rfloor }}(t)]\nonumber \\= & {} \sum _{j={\left\lfloor {a/\Delta }\right\rfloor }+1}^{\min \{{\left\lfloor {b/\Delta }\right\rfloor },{\left\lfloor {y/\Delta }\right\rfloor }\}}{{\left\lfloor {y/ \Delta }\right\rfloor }-1\atopwithdelims (){{\left\lfloor {y/ \Delta }\right\rfloor }}-j}\exp (-j\lambda t)(1-\exp (-\lambda t))^{{\left\lfloor {y/ \Delta }\right\rfloor }-j},\nonumber \\ \end{aligned}$$

where with respect to (9), we have used the correspondence $k={\left\lfloor {y/\Delta }\right\rfloor }$, $r={\left\lfloor {a/\Delta }\right\rfloor }$ and $s={\left\lfloor {b/\Delta }\right\rfloor }$. Note that $C_{\Delta }(y, t)$ is a piecewise constant function, with jumps at points of the form $k\Delta$. Furthermore, *k*, *r* and *s* increase at the same rate as $\Delta$ goes to zero, in particular, $r/k \rightarrow a/y$ and $s/k \rightarrow b/y$.

**Fig. 5:**
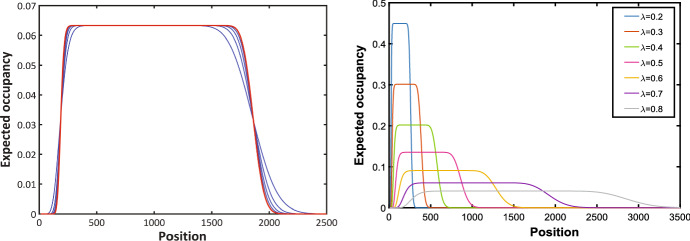
Left-hand panel: Evaluation of $C_{\Delta }(y, t)$ at $t = 4.0$ with initial particle mass on the interval [12, 118] $(\lambda = 0.69)$. Right-hand panel: $C_{\Delta }(y, t)$ plotted against different values of $\lambda$

For $t=4$ and an initial probability mass on the interval [12, 18], the blue lines in the left-hand panel of Fig. 4 depict $C_{\Delta }(y, t)$ as a function of *y* for values of $\Delta = 1/n$ with *n* varying from 1 to 10. Decreasing $\Delta$ corresponds to increasing the height of the curves and decreasing the tail mass. The right-hand panel of Fig. 4 shows $C_{\Delta }(y, t)$ as a function of $n=1/\Delta$ for $t=4$ and $y = 228$ (approximately where the peaks occur in the left hand panel of Fig. 5) for values of *n* up to 100. The blue lines in Fig. 5 provide another illustration of *y* plotted against $C_{\Delta }(y, 4)$ with 107 marked agents initially located at sites 12 up to 118, and $\Delta =1/n$ with *n* ranging from 1 to 5. We observe that, as $\Delta$ decreases, the curves become flatter with decreased tail mass.

### The limiting profile *C*(*y*, *t*)

Figures 4 and the left hand panel of 5 suggest that, taken as a function of *y* for a fixed value of *t*, $C_{\Delta }(y, t)$ approaches a limit as $\Delta \rightarrow 0$. We investigate this behaviour in this section, commencing with an intuitive characterisation using the normal approximation to the binomial distribution. Equation (14) for the expected number of particles at position *y* at time *t* when the spacing is $\Delta$ has the form

15 $$\begin{aligned} C_{\Delta }(y, t) = e^{-\lambda t} \Pr ({\left\lfloor {a/\Delta }\right\rfloor } \le S_{\Delta }(y,t)\le \min \{{\left\lfloor {b/\Delta }\right\rfloor }-1,{\left\lfloor {y/\Delta }\right\rfloor }-1\}), \end{aligned}$$

where $S_{\Delta }(y,t)$ is a random variable with a binomial distribution with parameters ${\left\lfloor {y/ \Delta }\right\rfloor }-1$ and $e^{-\lambda t}$. The mean and variance of $S_{\Delta }(y,t)$ are, respectively, $e^{-\lambda t}({\left\lfloor {y/ \Delta }\right\rfloor }-1)$ and $e^{-\lambda t}(1-e^{-\lambda t})({\left\lfloor {y/ \Delta }\right\rfloor }-1)$.

**Fig. 6:**
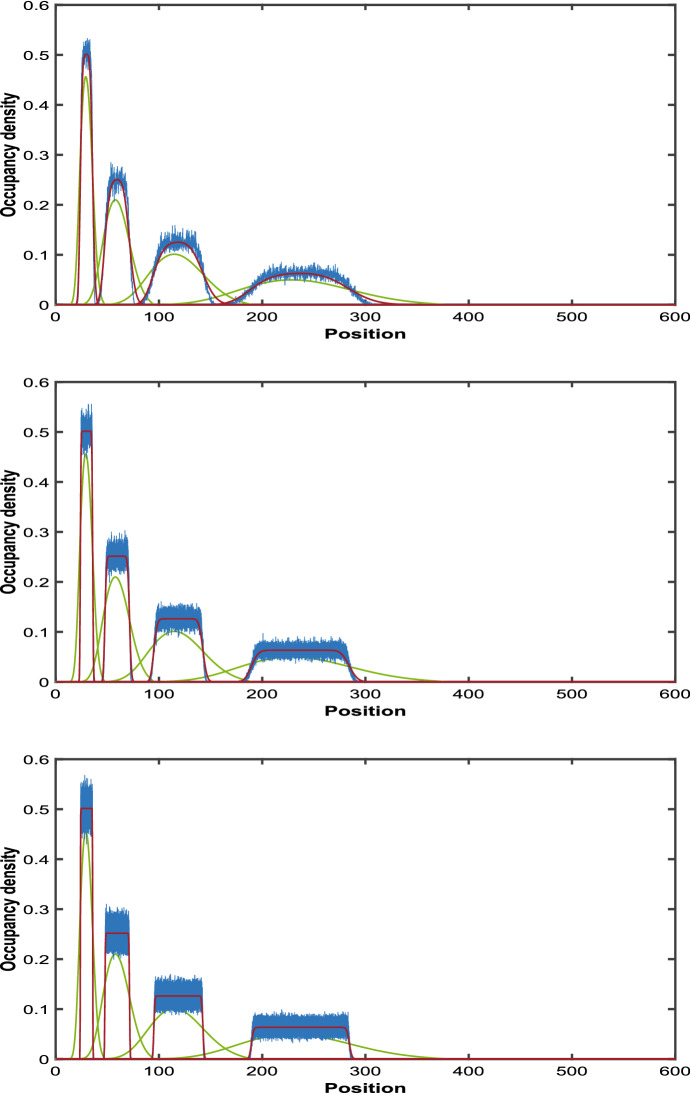
Expected occupancy averaged over 1000 realizations (blue), Solutions to Eq. (6) (green curve) and Occupation density (normal approximation (red)) with $\Delta = 0.1$, $\Delta =0.01$ and $\Delta =0.001$ at times $t = 1.0, 2.0, 3.0, 4.0,$ with initial particle mass on the interval [12, 18] and $\lambda = 0.69$

**Fig. 7:**
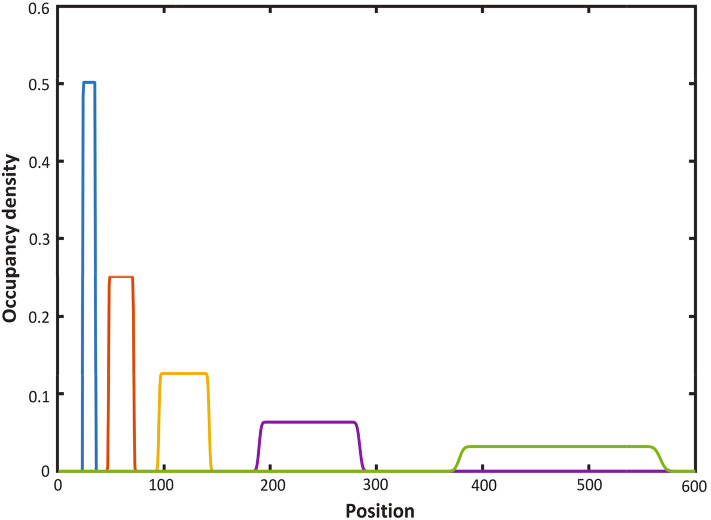
Occupation density (normal approximation with $\Delta = 0.001$ at times $t = 1.0, 2.0, 3.0, 4.0,$ and 5.0, with initial particle mass on the interval [12, 18] $(\lambda = 0.69)$

Employing the normal approximation to approximate $C_{\Delta }(y, t)$ when $\Delta$ is small, we get

16 $$\begin{aligned}&C_{\Delta }(y, t) \simeq C_{approx}(y, t) \nonumber \\&\quad = e^{-\lambda t} \Pr \left( \frac{a/y-e^{-\lambda t} }{\sqrt{e^{-\lambda t} (1-e^{-\lambda t} )}} \sqrt{{\left\lfloor {y/ \Delta }\right\rfloor }-1}\le Z\le \frac{\min (b/y,1)-e^{-\lambda t} }{\sqrt{e^{-\lambda t} (1-e^{-\lambda t} )}} \sqrt{{\left\lfloor {y/ \Delta }\right\rfloor }-1}\right) ,\nonumber \\ \end{aligned}$$

where *Z* is a standard normal random variable. The performance of this approximation is illustrated in red in the left hand panel of Fig. 4 and in the left-hand panel of Fig. 5. In our numerical studies, without loss of generality, we assume the value of $\lambda$ to be 0.69. We plotted $C_{\Delta }(y, t)$ for different values of $\lambda$ in the right hand panel of Fig. 5. In Fig. 6 we compare the normal approximation result in Eq. 16 with Eq. 6, derived by Hywood et al. (2013a). We see that Eq. 16 fits well with the simulation results averaged over 1000 of realizations and for $\Delta =0.1$, $\Delta =0.01$ and $\Delta =0.001$. Figure 7 illustrates how expression (16) varies with time.

The expression

$$\begin{aligned} \frac{a/y-e^{-\lambda t} }{\sqrt{e^{-\lambda t} (1-e^{-\lambda t} )}} \sqrt{{\left\lfloor {y/ \Delta }\right\rfloor }-1} , \end{aligned}$$

appearing in (16) is equal to zero if $a/y = e^{-\lambda t}$ and approaches $-\infty$ or $\infty$ as $\Delta \rightarrow 0$ depending on whether $a/y - e^{-\lambda t}$ is negative or positive, that is according as $y > ae^{\lambda t}$ or $y < ae^{\lambda t}$. Similarly,

$$\begin{aligned} \frac{\min (b/y,1)-e^{-\lambda t} }{\sqrt{e^{-\lambda t} (1-e^{-\lambda t} )}} \sqrt{{\left\lfloor {y/ \Delta }\right\rfloor }-1} , \end{aligned}$$

is equal to zero if $b/y = e^{-\lambda t}$ and, as $\Delta \rightarrow 0$, approaches $\infty$ if $y \le b$ or $b< y < b e^{\lambda t}$, and $-\infty$ otherwise. So

$$\begin{aligned} \Pr \left( \frac{a/y-e^{-\lambda t} }{\sqrt{e^{-\lambda t} (1-e^{-\lambda t} )}} \sqrt{{\left\lfloor {y/ \Delta }\right\rfloor }-1}\le Z\le \frac{\min \{b/y,1\}-e^{-\lambda t} }{\sqrt{e^{-\lambda t} (1-e^{-\lambda t} )}} \sqrt{{\left\lfloor {y/ \Delta }\right\rfloor }-1}\right) , \end{aligned}$$

approaches 1/2 if $y = a e^{\lambda t}$ or $b e^{\lambda t}$, 1 if $a e^{\lambda t}< y < b e^{\lambda t}$ and 0 otherwise. Thus we would expect the limiting profile for $C_{\Delta }(y, t)$ to be

17 $$\begin{aligned} C(y, t):=\lim _{\Delta \rightarrow 0} C_{\Delta }(y,t) = {\left\{ \begin{array}{ll} 0 &{}\text {if } 0<y< ae^{\lambda t} \\ \frac{1}{2} e^{-\lambda t} &{}\text {if } y=ae^{\lambda t} \\ e^{-\lambda t} &{}\text {if } ae^{\lambda t}< y < be^{\lambda t} \\ \frac{1}{2} e^{-\lambda t} &{}\text {if } y=be^{\lambda t} \\ 0 &{}\text {if } y > be^{\lambda t}. \end{array}\right. } \end{aligned}$$

#### Remark 4.1

Note that *C*(*y*, *t*), given by (17)

- has the correct mass $b-a$ for all *t*, and
- is a weak solution of the first-order partial differential Eq. (3).

It remains to establish the form (17) rigorously as $\Delta \rightarrow 0$. This is given in the following theorem.

#### Theorem 4.1

With the spacing set at $\Delta$, let $C_{\Delta }(y, t) = E[X_{{\left\lfloor {y/ \Delta }\right\rfloor }} (t)]$ be the expected number of particles at site ${\left\lfloor {y/ \Delta }\right\rfloor }$ at time *t*.

(i) For all positive *y* and *t*, $\lim _{\Delta \rightarrow 0} C_{\Delta }(y,t)$ is given by (17).
(ii) For all positive *y* and *t*, $\lim _{\Delta \rightarrow 0} V[X_{{\left\lfloor {y/ \Delta }\right\rfloor }} (t)] = C(y, t)(1-C(y, t))$ where *C*(*y*, *t*) is defined in (17).
(iii) For $w \in (a,b]$ and all positive *t*, let $Z_{\Delta }(w,t) = \Delta Z^{{\left\lfloor {w/\Delta }\right\rfloor }}(t)$ be the separation at time *t* of the agents that start at $\Delta {\left\lfloor {w/\Delta }\right\rfloor }$ and $\Delta ({\left\lfloor {w/\Delta }\right\rfloor } + 1)$. Then, for all $u>0$, $\lim _{\Delta \rightarrow 0} \Pr (Z_{\Delta }(w,t) >u) = 0$.

#### Proof

See the Appendix. $\square$

We started this section by observing that we can think of our initial condition as spreading a mass of $\Delta {\left\lfloor {(b-a)/\Delta }\right\rfloor }$ over $N(0) = {\left\lfloor {(b-a)/\Delta }\right\rfloor }$ points, so that each point carries a mass of $\Delta$. In the limit as $\Delta \rightarrow 0$ the mass at each point goes to zero but the number of points approaches infinity in such a way that the total mass approaches $b-a$.

For $y \in (a,\infty )$, this motivates us to define $M_\Delta (y,t) = \Delta M_{{\left\lfloor {y/\Delta }\right\rfloor }} (t)$ in the model where the spacing is set to $\Delta$ and $M_k(t)$ is defined in part (iv) of Theorem 3.1. The limiting distribution of $M_\Delta (y,t)$ is given in the following theorem.

#### Theorem 4.2

(i) For positive *t*, $y \in (a,\infty )$ and $z \in (a,b)$,
  18 $$\begin{aligned} \lim _{\Delta \rightarrow 0} \Pr (M_\Delta (y,t) \le z - a ) = {\left\{ \begin{array}{ll} 1 &{}\text {if } z \ge ye^{-\lambda t} \\ 0 &{}\text {if } z < ye^{-\lambda t}. \end{array}\right. } \end{aligned}$$
(ii) For positive *t*, $y \in (ae^{\lambda t},be^{\lambda t}]$ and $z \in (-\infty ,\infty )$,
  19 $$\begin{aligned} \lim _{\Delta \rightarrow 0} \Pr \left( \frac{M_\Delta (y,t) - \Delta ({\left\lfloor {y e^{-\lambda t}/\Delta }\right\rfloor } - {\left\lfloor {a/\Delta }\right\rfloor })}{\Delta \sqrt{(e^{-\lambda t} - e^{-2\lambda t}) {\left\lfloor {y/\Delta }\right\rfloor }}} \le z \right) = \Phi (z), \end{aligned}$$
  where $\Phi (z) = \int _{-\infty }^z e^{-u^2/2}/\sqrt{2\pi }du$ is the standard normal distribution function.

#### Proof

See the Appendix. $\square$

#### Remark 4.2

If $y<ae^{\lambda t}$, then Theorem 4.2(i) implies that $\lim _{\Delta \rightarrow 0} \Pr (M_\Delta (y,t) \le z - a ) = 1$ for all $z \in (a,b]$. In particular, this implies that $\lim _{\Delta \rightarrow 0} \Pr (M_\Delta (y,t) \le 0 ) = 1$, which tells us that $M_\Delta (y,t)$ weakly converges to a point mass at zero. On the other hand, if $y>be^{\lambda t}$ then $\lim _{\Delta \rightarrow 0} \Pr (M_\Delta (y,t) \le z - a ) = 0$ for all $z \in (a,b)$ and $M_\Delta (y,t)$ weakly converges to a point mass at $b-a$. Otherwise, $M_\Delta (y,t)$ weakly converges to a point mass at $z=ye^{-\lambda t} - a$.

This agrees with the observation of Theorem 4.1 that the limiting expected number of agents *C*(*y*, *t*) has the form of a square wave of height $e^{-\lambda t}$ over the interval $y \in (ae^{\lambda t},be^{\lambda t})$.

## Conclusion

In this paper we have analysed the cellular proliferation process model of Hywood et al. (2013a) by taking advantage of its relationship to the superposition of a set of coupled Yule-Furry processes, each with the same splitting rate. In Sect. 2, we presented the model of Hywood et al. (2013a) together with the argument of Hywood et al. (2013a) to derive an approximating second order PDE for the expected occupancy of the process at position *y* at time *t*.

In Sect. 3, we analysed the discrete model with positive $\Delta$, and used the model’s representation as a set of coupled Yule-Furry processes to derive an exact expression for the expected occupancy $C_{k}(t$) at site *k* at time *t*. We also derived expressions for the variance of the occupancy and the distribution of the number of sites between adjacent domain agents. Finally, we showed that at time *t*, domain agents occupy positions that are distributed as a geometric renewal process. In Sect. 4, we considered limiting versions as $\Delta \rightarrow 0$ of the process that we analysed in Sect. 3. Theorem 4.1 contains expressions for the limiting expected occpuancy at position *y* at time *t* and its variance, while Theorem 4.2 presents Weak Law of Large Numbers and Central Limit Theorem results for the total mass of domain agents occupying positions to the left of *y* at time *t*.

## Appendix

### Proof of Theorm 3.1

(i) The first assertion follows from the argument outlined in Sect. 3: the expected number of particles at site *k* is the same in the proliferation process as it is in the asymmetric random walk process, which is given by (9).
(ii) The number of points at site *k* in the proliferation process is a Bernoulli random variable with a probability $C_k(t)$ of being equal to one. It follows that its variance is equal to $C_k(t)(1-C_k(t))$.
(iii) Using our view of the Yule-Furry process as being generated by a sequence of independent homogeneous Poisson processes $\{{{\mathcal {N}}}_{i}\}$ with rate $\lambda$ at each integer point *i*, we see that the distance $Z^{(j)}$ between two marked domain agents also grows as a Yule-Furry process, but one that starts off with $Z^{(j)}(0) = 1$. Equation (13) then follows from Eq. (7).
(iv) If $k \le j$, then the proliferation process started with less than or equal to $j-r$ agents in positions $r+1,\ldots ,k$ and, since agents never move to lower numbered positions, $\Pr (M_k(t) \le j-r) = 1$ for all *t*.
  For $k > j$, the event that there are less than $j-r$ marked agents in positions $r+1,\ldots ,k$ at time *t* is the same as the event that the agent starting in position *j* has moved to one of the positions $\ell > k$ at time *t*. From this observation, it follows that $\Pr (M_k(t) \le j-r) = \Pr (M_k(t) < j-r+1) = \Pr (Y^{(j+1)}(t)> k)$. Substituting Eq. (7) we see that, for $j=r+1, \ldots , s$,
  20 $$\begin{aligned}&\Pr (M_k(t) \le j-r)\nonumber \\&\quad = \sum _{\ell = k+1} ^\infty {{\ell -1}\atopwithdelims (){\ell -j-1}} e^{-(j+1)\lambda t}\left( 1-e^{-\lambda t}\right) ^{\ell -j-1}\nonumber \\&\quad = e^{-(j+1)\lambda t} \sum _{\ell = 0}^\infty {{\ell +k}\atopwithdelims (){\ell +k-j}} \left( 1-e^{-\lambda t}\right) ^{\ell +k-j}\nonumber \\&\quad = \frac{e^{-(j+1)\lambda t}\left( 1-e^{-\lambda t}\right) ^{k-j}}{j!} \sum _{\ell = 0}^\infty \frac{(\ell +k)!\ \ell !}{(\ell +k-j)!} \frac{\left( 1-e^{-\lambda t}\right) ^{\ell }}{\ell !}\nonumber \\&\quad = {k\atopwithdelims ()j} e^{-(j+1)\lambda t}\left( 1-e^{-\lambda t}\right) ^{k-j}\ _2F_1(k+1,1;k-j+1;1-e^{-\lambda t}),\nonumber \\ \end{aligned}$$
  by (Abramowitz and Stegun 1965, Definition 15.1.1).

$$\square $$

### Proof of Theorem 4.1

For sequences $(a_i)$ and $(b_i)$, we shall write $a_i \sim b_i$ if $\limsup _{i\rightarrow \infty } |a_i/b_i|=1$.

Our proof involves the Kullback-Liebler divergence (or *relative entropy*)

21 $$\begin{aligned} H(\epsilon ,\theta ) = \epsilon \log \left( \frac{\epsilon }{\theta } \right) + (1-\epsilon ) \log \left( \frac{1-\epsilon }{1-\theta } \right) , \end{aligned}$$

between the Bernoulli distribution with success probability $\epsilon$ and the Bernoulli distribution with success probability $\theta$.

Suppose $S_k$ has a binomial $B(k,\theta )$ distribution. Then, by Theorem 2 of Arratia and Gordon (1989), if $\theta<\epsilon <1$,

22 $$\begin{aligned} \Pr (S_k \ge k\epsilon ) \sim \frac{1}{(1-r)\sqrt{2\pi \epsilon (1-\epsilon ) k}} e^{-kH(\epsilon ,\theta )}, \end{aligned}$$

where

23 $$\begin{aligned} r=r(\epsilon ,\theta )=\left( \frac{\theta }{1-\theta }\right) \Big /\left( \frac{\epsilon }{1-\epsilon }\right) =\frac{\theta (1-\epsilon )}{\epsilon (1-\theta )}. \end{aligned}$$

We can deal with the complementary case where $0<\epsilon <\theta$ by noting that $k-S_k$ has a binomial $B(k,1-\theta )$ distribution. Clearly $H(1-\epsilon ,1-\theta )=H(\epsilon ,\theta )$, and $r(1-\epsilon ,1-\theta )=1/r(\epsilon ,\theta )$ $(<1)$, and so

24 $$\begin{aligned} \Pr (S_k \le k\epsilon ) =\Pr (k-S_k \ge i(1-\epsilon )) \sim \frac{1}{(1-1/r)\sqrt{2\pi \epsilon (1-\epsilon ) k}} e^{-kH(\epsilon ,\theta )}.\nonumber \\ \end{aligned}$$

Finally, when $\epsilon = \theta$, the Central Limit Theorem tells us that

25 $$\begin{aligned} \lim _{k\rightarrow \infty } \Pr (S_k \ge k\theta )=1/2. \end{aligned}$$

We proceed to the proof of the theorem.

(i) Referring to (15), let $\Xi _{\Delta }(y,t)= e^{\lambda t} C_{\Delta }(y, t)$.
  There are two main cases to consider. First, when $y<b$, $\Xi _{\Delta }(y,t) = \Pr (S_{\Delta }(y) > {\left\lfloor {a/\Delta }\right\rfloor })$. With reference to Eqs. (21) and (23), we take $\theta = e^{-\lambda t}$ and $\epsilon = a/y$ so that, in the obvious notation,
  26 $$\begin{aligned} r(a,y,t) =\frac{e^{-\lambda t} (y-a)}{a(1- e^{-\lambda t})}, \end{aligned}$$
  and
  27 $$\begin{aligned} H(a,y,t) = (a/y) \log \left( \frac{a}{ye^{-\lambda t}} \right) + ((y-a)/y) \log \left( \frac{y-a}{y(1-e^{-\lambda t})} \right) . \end{aligned}$$
  Then, from Eq. (22), if $e^{-\lambda t}< a/y <1$,
  $$\begin{aligned}&\Xi _{\Delta }(y,t) \\&\quad \sim \frac{a(1-e^{-\lambda t})\sqrt{y\Delta }}{(a-ye^{-\lambda t})\sqrt{2\pi a(y-a)}} \\&\qquad \exp \left[ -(a/\Delta ) \log \left( \frac{a}{ye^{-\lambda t}} \right) - ((y-a)/\Delta ) \log \left( \frac{y-a}{y(1-e^{-\lambda t})} \right) \right] , \end{aligned}$$
  which approaches 0 as $\Delta \rightarrow 0$. Alternatively, from (24), if $a/y<e^{-\lambda t}$, then
  $$\begin{aligned}&\Xi _{\Delta }(y,t) \\&\quad \sim 1-\frac{(y-a)e^{-\lambda t}\sqrt{y\Delta }}{(ye^{-\lambda t}-a)\sqrt{2\pi a(y-a)}}\\&\qquad \exp \left[ -(a/\Delta ) \log \left( \frac{a}{ye^{-\lambda t}} \right) - ((y-a)/\Delta ) \log \left( \frac{y-a}{y(1-e^{-\lambda t})} \right) \right] , \end{aligned}$$
  which approaches 1 as $\Delta \rightarrow 0$. We deduce that, if $ae^{\lambda t}<y<b$, then $\Xi _{\Delta }(y,t) \rightarrow 1$. On the other hand, if $y < \min (ae^{\lambda t},b)$ then $\Xi _{\Delta }(y,t) \rightarrow 0$ and if $y=ae^{\lambda t} < b$, then $\Xi _{\Delta }(y,t) \rightarrow 1/2$.
  Now consider the case where $y\ge b$ and so
  28 $$\begin{aligned} \Xi _{\Delta }(y,t) = \Pr ({\left\lfloor {a/\Delta }\right\rfloor } +1 \le S_\Delta (y) \le {\left\lfloor {b/\Delta }\right\rfloor }). \end{aligned}$$
  If $e^{-\lambda t} \ge b/y > a/y$, (28) gives us
  $$\begin{aligned}&\Xi _{\Delta }(y,t) \\&\quad \sim \frac{(y-b)e^{-\lambda t}\sqrt{y\Delta }}{(ye^{-\lambda t}-b)\sqrt{2\pi b(y-b)}}\\&\qquad \exp \left[ -(b/\Delta ) \log \left( \frac{b}{ye^{-\lambda t}} \right) - ((y-b)/\Delta ) \log \left( \frac{y-b}{y(1-e^{-\lambda t})} \right) \right] \\&\qquad -\frac{(y-a)e^{-\lambda t}\sqrt{y\Delta }}{(ye^{-\lambda t}-a)\sqrt{2\pi a(y-a)}}\\&\qquad \exp \left[ -(a/\Delta ) \log \left( \frac{a}{ye^{-\lambda t}} \right) - ((y-a)/\Delta ) \log \left( \frac{y-a}{y(1-e^{-\lambda t})} \right) \right] , \end{aligned}$$
  implying that, for all such *y*, $\Xi _{\Delta }(y,t) \rightarrow 0$.
  If $$e^{-\lambda t}< a/y <b/y$$,
  $$\begin{aligned}&\Xi _{\Delta }(y,t) \\&\quad \sim \frac{a(1-e^{-\lambda t})\sqrt{y\Delta }}{(a-ye^{-\lambda t})\sqrt{2\pi a(y-a)}} \\&\qquad \exp \left[ -(a/\Delta ) \log \left( \frac{a}{ye^{-\lambda t}} \right) - ((y-a)/\Delta ) \log \left( \frac{y-a}{y(1-e^{-\lambda t})} \right) \right] \\&\qquad -\frac{b(1-e^{-\lambda t})\sqrt{y\Delta }}{(b-ye^{-\lambda t})\sqrt{2\pi b(y-b)}} \\&\qquad \exp \left[ -(b/\Delta ) \log \left( \frac{b}{ye^{-\lambda t}} \right) - ((y-b)/\Delta ) \log \left( \frac{y-b}{y(1-e^{-\lambda t})} \right) \right] , \end{aligned}$$
  implying that $\Xi _{\Delta }(y,t) \rightarrow 0$ in this case as well.
  If $$a/y< e^{-\lambda t} < b/y$$
  $$\begin{aligned}&\Xi _{\Delta }(y,t) \\&\quad \sim 1 - \frac{b(1-e^{-\lambda t})\sqrt{y\Delta }}{(b-ye^{-\lambda t})\sqrt{2\pi b(y-b)}} \\&\qquad \exp \left[ -(b/\Delta ) \log \left( \frac{b}{ye^{-\lambda t}} \right) - ((y-b)/\Delta ) \log \left( \frac{y-b}{y(1-e^{-\lambda t})} \right) \right] \\&\qquad -\frac{(y-a)e^{-\lambda t}\sqrt{y\Delta }}{(ye^{-\lambda t}-a)\sqrt{2\pi a(y-a)}}\\&\qquad \exp \left[ -(a/\Delta ) \log \left( \frac{a}{ye^{-\lambda t}} \right) - ((y-a)/\Delta ) \log \left( \frac{y-a}{y(1-e^{-\lambda t})} \right) \right] , \end{aligned}$$
  and, in this case, $\Xi _{\Delta }(y,t) \rightarrow 1$.
  Finally, if $e^{-\lambda t} = a/y$ or $e^{-\lambda t} = b/y$, then $\Xi _{\Delta }(y,t) \rightarrow 1/2$ because the inequality on one side of (28) is satisfied in the limit with probability 1/2. This proves (i).
(ii) For any finite value of $\Delta$, the number of particles at location *y*, that is $X_{{\left\lfloor {y/\Delta }\right\rfloor }}$, is a Bernoulli random variable: there is either a particle at site ${\left\lfloor {y/\Delta }\right\rfloor }$ or there isn’t. Since the mean of this Bernoulli random variable is $C_{\Delta }(y,t)$, its variance must be $Var_{\Delta }(y,t) =C_{\Delta }(y,t)(1-C_{\Delta }(y,t))$. This proves (ii).
(iii) For $$w \in (a,b]$$,
  $$\begin{aligned} \lim _{\Delta \rightarrow 0} \Pr (Z_{\Delta }(w,t)>u)= & {} \lim _{\Delta \rightarrow 0}\Pr (Z^{{\left\lfloor {w/\Delta }\right\rfloor }}(t) > u/\Delta )\nonumber \\= & {} \lim _{\Delta \rightarrow 0} \sum _{\ell = {\left\lfloor {u/\Delta }\right\rfloor }+1}^\infty e^{-\lambda t}\left( 1-e^{-\lambda t}\right) ^{\ell -1}\nonumber \\= & {} \lim _{\Delta \rightarrow 0} \left( 1-e^{-\lambda t}\right) ^{{\left\lfloor {u/\Delta }\right\rfloor }}\nonumber \\= & {} 0, \end{aligned}$$
  where the second equation follows from (13).

$$\square $$

### Proof of Theorem 4.2

(i) If $y \le z$, it follows from part (iv) of Theorem 3.1 that
  $$\begin{aligned} \Pr \left( M_{\Delta } (y,t) \le \Delta ({\left\lfloor {z/\Delta }\right\rfloor } - {\left\lfloor {a/\Delta }\right\rfloor })\right) = 1, \end{aligned}$$
  for all $\Delta$ and so $\lim _{\Delta \rightarrow 0}\Pr \left( M_{\Delta } (y,t) \le z-a\right) = 1$. For $y>z$, (20) gives us
  29 $$\begin{aligned}&\Pr \left( M_{\Delta } (y,t) \le \Delta ({\left\lfloor {z/\Delta }\right\rfloor } - {\left\lfloor {a/\Delta }\right\rfloor })\right) \nonumber \\&\quad = \sum _{\ell = {\left\lfloor {y/\Delta }\right\rfloor }+1}^\infty {{\ell -1}\atopwithdelims (){\ell -{\left\lfloor {z/\Delta }\right\rfloor }-1}} e^{- ({\left\lfloor {z/\Delta }\right\rfloor }+1)\lambda t}\left( 1-e^{-\lambda t}\right) ^{\ell - {\left\lfloor {z/\Delta }\right\rfloor }-1}. \end{aligned}$$
  The summand on the right hand side of (29) is $\Pr (R({\left\lfloor {z/\Delta }\right\rfloor } +1,t)=\ell )$ where *R*(*n*, *t*) is a negative binomial random variable with parameters *n* and $e^{-\lambda t}$. The right hand side of (29) itself can thus be interpreted as $\Pr \left( R({\left\lfloor {z/\Delta }\right\rfloor } +1,t)\ge {\left\lfloor {y/\Delta }\right\rfloor }+1\right)$. The random variable *R*(*n*, *t*) has the same distribution as the sum $\sum _{i=1}^{n}T^{(i)}(t)$ where $T^{(i)}(t)$ are independent geometric random variables with common parameter $e^{-\lambda t}$. The mean of $T^{(i)}(t)$ is $e^{\lambda t}$ and its variance is $e^{2\lambda t} - e^{\lambda t}$. We can apply the Weak Law of Large Numbers to conclude that the distribution of $R({\left\lfloor {z/\Delta }\right\rfloor } +1,t)/({\left\lfloor {z/\Delta }\right\rfloor }+1)$ approaches a point mass at $e^{\lambda t}$ as $\Delta \rightarrow 0$. For $z \in (a,\min (y,b)]$, we conclude that
  $$\begin{aligned}&\lim _{\Delta \rightarrow 0}\Pr \left( M_{\Delta } (y,t) \le \Delta ({\left\lfloor {z/\Delta }\right\rfloor } - {\left\lfloor {a/\Delta }\right\rfloor })\right) \\&\quad = \lim _{\Delta \rightarrow 0} \Pr \left( R({\left\lfloor {z/\Delta }\right\rfloor } +1,t)\ge {\left\lfloor {y/\Delta }\right\rfloor }+1\right) \\&\quad = \lim _{\Delta \rightarrow 0} \Pr \left( \frac{R({\left\lfloor {z/\Delta }\right\rfloor } +1,t)}{{\left\lfloor {z/\Delta }\right\rfloor }+1} \ge \frac{{\left\lfloor {y/\Delta }\right\rfloor }+1}{{\left\lfloor {z/\Delta }\right\rfloor }+1}\right) \\&\quad = {\left\{ \begin{array}{ll} 1 &{}\text {if } {\displaystyle \frac{y}{z} \le e^{\lambda t}} \\ \\ 0 &{}\text {if } {\displaystyle \frac{y}{z} > e^{\lambda t},} \end{array}\right. } \end{aligned}$$
  and the result is proved.
(ii) Let
  $$\begin{aligned} \Upsilon _{\Delta }(y,t) = \frac{M_\Delta (y,t) - \Delta ({\left\lfloor {y e^{-\lambda t}/\Delta }\right\rfloor } - {\left\lfloor {a/\Delta }\right\rfloor })}{\Delta \sqrt{(e^{-\lambda t} - e^{-2\lambda t}) {\left\lfloor {y/\Delta }\right\rfloor }}}. \end{aligned}$$
  Then, by (20),
  30 $$\begin{aligned}&\lim _{\Delta \rightarrow 0} \Pr (\Upsilon _{\Delta }(y,t) \le z)\nonumber \\&\quad = \lim _{\Delta \rightarrow 0}\Pr \bigg (M_\Delta (y,t) \le \Delta \bigg (({\left\lfloor {y e^{-\lambda t}/\Delta }\right\rfloor } - {\left\lfloor {a/\Delta }\right\rfloor })\nonumber \\&\qquad \left. \left. + z \sqrt{(e^{-\lambda t} - e^{-2\lambda t}) {\left\lfloor {y/\Delta }\right\rfloor }}\right) \right) \nonumber \\&\quad = \lim _{\Delta \rightarrow 0}\Pr (R(n(\Delta )) > {\left\lfloor {y/\Delta }\right\rfloor }), \end{aligned}$$
  where $n(\Delta ) = {\left\lfloor {{\left\lfloor {y e^{-\lambda t}/\Delta }\right\rfloor } + z \sqrt{(e^{-\lambda t} - e^{-2\lambda t}) {\left\lfloor {y/\Delta }\right\rfloor }}}\right\rfloor }$. The limiting probability (30) is the same as
  31 $$\begin{aligned}&\lim _{\Delta \rightarrow 0} \Pr \left( \frac{R(n(\Delta )) - n(\Delta )e^{\lambda t}}{\sqrt{n(\Delta )(e^{2\lambda t} - e^{\lambda t})}}> \frac{{\left\lfloor {y/\Delta }\right\rfloor }- n(\Delta )e^{\lambda t}}{\sqrt{n(\Delta )(e^{2\lambda t} - e^{\lambda t})}}\right) \nonumber \\&\quad = \lim _{\Delta \rightarrow 0} \Pr \left( \frac{R(n(\Delta )) - n(\Delta )e^{\lambda t}}{\sqrt{n(\Delta )(e^{2\lambda t} - e^{\lambda t})}} \right. \nonumber \\&\quad \left. > \frac{- z \sqrt{(e^{\lambda t} - 1) {\left\lfloor {y/\Delta }\right\rfloor }}}{\sqrt{(e^{\lambda t} - 1) {\left\lfloor {y/\Delta }\right\rfloor } + z(e^{2\lambda t} - e^{\lambda t})\sqrt{(e^{-\lambda t} - e^{-2\lambda t}) {\left\lfloor {y/\Delta }\right\rfloor }}}}\right) \nonumber \\&\quad = \Phi (z), \end{aligned}$$
  by the Central Limit Theorem and because the expression on the right hand side approaches $-z$ as $\Delta \rightarrow 0$.

$$\square $$
